# A Submarine Thermometer

**Published:** 1843-04

**Authors:** 


*A Submarine Thermometer.*—This is a French invention of the celebrated Mons. Clement, which has been tried lately in England in presence of the Lords of the Admiralty, on board the Lightning (steam vessel) Lieutenant Commander G. Snell. In relation to this new invention, we find the following in a late London paper.

It appears from the thermometrical observations of many scientific navigators, that in seas of unfathomable depth the water is not so cold as over the banks, and that over banks near the shore it is less cold than over those at a greater distance, but colder than in the sea. In experiments which have been made on the coast of France with this submarine thermometer results have been obtained which fully establish the great service which this instrument may render to navigation, by furnishing a sure and constant indication of all sudden changes from deep to shallow water. It is evident also that it will serve to point out the proximity of vessels to icebergs which at certain seasons of the year render the navigation of the Atlantic attended with danger. This test could not, of course, be made in the experiments with the Lightning, but the other important qualities are correctly ascertained. Clement's thermometer is kept constantly under water at the same depth, and indicates the different temperatures of the water by means of a dial placed on the deck of the vessel, and always open to examination. The immediate action is communicated by wheels, the working of which turns two hands upon the dial, the one marking the single degrees, and the other the tens. The whole is enclosed in a tube, and attached to the side of the vessel.

The same gentlema, Clement, is also the inventor of two instruments that have been well tested in France and England, to ascertain the speed of steam vessels, and to determine the heat of water in their boilers. The first is named a *Sillometer*, a title given to a substitute for the common log; and the second is termed the *Steam Engine Indicator.* The three instruments are described as being inventions of great practical value.

